# Draft genome sequences of five endophytic fungi isolated from *Lactuca serriola*, a wild relative of cultivated lettuce

**DOI:** 10.1128/mra.00444-25

**Published:** 2025-10-07

**Authors:** Neda Arad, Joseph Spraker, Kayla Garcia, Duke Pauli, A. Elizabeth Arnold

**Affiliations:** 1 School of Plant Sciences, The University of Arizona 8041, Tucson, Arizona, USA; 2 Ecosystem Genomics Graduate Interdisciplinary Program, The University of Arizona 8041, Tucson, Arizona, USA; 3 Hexagon Bio 542226 , Menlo Park, California, USA; 4 Center for Agroecosystem Research in the Desert (ARID), The University of Arizona 8041, Tucson, Arizona, USA; 5 BIO5 Institute, The University of Arizona 8041, Tucson, Arizona, USA; 6 Department of Ecology and Evolutionary Biology, The University of Arizona 8041, Tucson, Arizona, USA; University of California Riverside, Riverside, California, USA

**Keywords:** endophytes, fungi, genome analysis

## Abstract

We present draft genome sequences of five endophytic fungi from *Lactuca serriola* L., a wild relative of cultivated lettuce: *Alternaria postmessia*, two *Alternaria alternata* variants, *Fusarium falciforme*, and *Aspergillus terreus*. Isolates were obtained from field-grown plants in Arizona, USA, and whole-genome sequenced using Illumina NovaSeq 6000 at ≥50× coverage.

## ANNOUNCEMENT

Endophytic fungi are microorganisms that live within plant tissues without causing disease symptoms (1). They can influence plant growth, stress tolerance, and disease resistance (1, 2). *Lactuca serriola*, a wild relative of cultivated lettuce, harbors diverse fungal endophytes, some of which can enhance the quality of cultivated lettuce when applied as microbial inocula (3). Their genomic properties remain largely unexplored.

We sequenced the genomes of five endophytic fungi isolated from surface-sterilized stems and roots of *L. serriola* to characterize their genomic features. Whole plants were dug out by hand to collect healthy roots and stems at field margins and roadsides adjacent to fields at the Maricopa Agricultural Center, AZ, USA (4). The isolates were identified as *Alternaria postmessia* from the root, *Alternaria alternata* from the root (strain 1) and stem (strain 2), *Fusarium falciforme* from the root, and *Aspergillus terreus* from the root (Table 1). These genera include common endophytes of cultivated lettuce in the study area (4). Vouchers of each culture are maintained at the University of Arizona’s Robert L. Gilbertson Mycological Herbarium. For taxonomic identification, assembled sequences were compared against the National Center for Biotechnology Information (NCBI) fungal genome database using the BLASTn version 2.2.31+ algorithm under default parameters, with top-scoring alignments used for species assignment.

**TABLE 1:** Summary of genome assembly statistics for five endophytic fungi isolated from *L. serriola*

Fungal isolate	Accession	NCBI assembly	Source	Medium	Genome size (Mb)	GC content (%)	No. of genes	Coverage	Contigs	N50 (bp)	Average read length (bp)	Total reads (bp)	Genome completeness (%)
*A. postmessia*	AEH-0273	GCA_049724655.1	Root of *L. serriola*	Malt extract agar (MEA)	34.7	51	13,488	217×	2,251	1,302,337	151	49,876,200	99.3
*A. alternata*	AEH-3014	GCA_049724695.1	Root of *L. serriola*	Potato dextrose agar	34.3	51	13,137	138.6×	910	789,874	151	31,225,294	99.3
*A. alternata*	AEH-3049	GCA_049724635.1	Stem of *L. serriola*	MEA	35.1	51	13,351	171.5×	5,217	1,066,199	151	39,558,194	98.9
*F. falciforme*	UA-600A	GCA_049863005.1	Root of *L. serriola*	MEA	54.2	50	15,174	118.2×	2,328	501,434	151	42,130,560	99.7
*A. terreus*	UA-600B	GCA_049862995.1	Root of *L. serriola*	MEA	30.6	52	10,703	91.1×	552	505,116	151	18,373,652	99.5

The strains were grown in a static medium (Table 1) for 14 days at 25°C. Mycelium was freeze-dried at −80°C, lyophilized, and pulverized via bead beating. Genomic DNA was purified from ground mycelium with a Zymobiomics Soil Kit (Zymo Research Corporation, Irvine, CA, USA). For preparation of sequencing libraries, 25 ng of total genomic DNA were used as the template and processed using the KAPA HyperPlus Kit for PCR-free workflows (Roche, Switzerland) followed by eight rounds of amplification to increase library yields. Sequencing libraries were pooled and size selected for 350–900 bp fragments using Zymo Select-a-size Clean & Concentrator and gel purification. Whole genomes were sequenced on the NoveSeq 6000 platform (Illumina, San Diego, CA, USA) with ≥50× coverage. Prior to assembly, adapters were trimmed, and low-quality reads were filtered with fastp v 0.21.0 (5). Genomes were assembled using SPAdes v 3.15.4 (6) with standard parameters. Annotations were carried out using BRAKER2 v 2.1.2 (7) with protein models from the closest relatives available in the Joint Genome Institute database. No RNA-seq reads were used to inform the BRAKER2 runs. Secondary metabolite biosynthetic gene clusters (BGCs) were identified by analysis in antiSMASH v 7.1.0 (8).

Genome assembly statistics for the five endophytic fungi isolated from *L. serriola* are summarized in Table 1. Data on BGCs and BRAKER2 annotation output files (.gff) are available at Zenodo: https://doi.org/10.5281/zenodo.15320263. Although these BGCs are not characterized here, several show a high similarity to those for known compounds and mycotoxins, like sansalvamide, which may impact interactions with host plants or other microorganisms. Future work should test them *in vivo* and assess their potential occurrence in cultivated lettuce.

## Data Availability

Sequence data from these genomes are available at GenBank under BioProject accession number PRJNA1241247. Individual genome assemblies are available under accession numbers GCA_049724695.1, GCA_049724635.1, GCA_049724655.1, GCA_049862995.1, and GCA_049863005.1. Raw sequencing reads are available in the Sequence Read Archive under accession numbers SRR33476769, SRR33476768, SRR33476767, SRR33476766, and SRR33476765. Additional data on BGC are available at Zenodo: https://doi.org/10.5281/zenodo.15320263.
